# The Effect of an Elevated Dietary Copper Level on the Vascular Contractility and Oxidative Stress in Middle-Aged Rats

**DOI:** 10.3390/nu16081172

**Published:** 2024-04-15

**Authors:** Klaudia Kitala-Tańska, Katarzyna Socha, Jerzy Juśkiewicz, Magdalena Krajewska-Włodarczyk, Michał Majewski

**Affiliations:** 1 Department of Pharmacology and Toxicology, Faculty of Medicine, University of Warmia and Mazury in Olsztyn, 10-082 Olsztyn, Poland; michal.majewski@uwm.edu.pl; 2 Department of Bromatology, Medical University of Białystok, 15-222 Białystok, Poland; katarzyna.socha@umb.edu.pl; 3 Division of Food Science, Institute of Animal Reproduction and Food Research, Polish Academy of Sciences, 10-748 Olsztyn, Poland; j.juskiewicz@pan.olsztyn.pl; 4 Department of Mental and Psychosomatic Diseases, Faculty of Medicine, University of Warmia and Mazury in Olsztyn, 10-082 Olsztyn, Poland; magdalenakw@op.pl

**Keywords:** copper, AH-6809, AL-8810, NS-398, TCP

## Abstract

Copper (Cu), being an essential mineral, plays a crucial role in maintaining physiological homeostasis across multiple bodily systems, notably the cardiovascular system. However, an increased Cu level in the body may cause blood vessel dysfunction and oxidative stress, which is unfavorable for the cardiovascular system. Middle-aged (7–8 months old) male Wistar rats (n/group = 12) received a diet supplemented with 6.45 mg Cu/kg (100% of the recommended daily dietary quantity of copper) for 8 weeks (Group A). The experimental group received 12.9 mg Cu/kg of diet (200%—Group B). An ex vivo study revealed that supplementation with 200% Cu decreased the contraction of isolated aortic rings to noradrenaline (0.7-fold) through FP receptor modulation. Vasodilation to sodium nitroprusside (1.10-fold) and acetylcholine (1.13-fold) was potentiated due to the increased net effect of prostacyclin derived from cyclooxygenase-1. Nitric oxide (NO, 2.08-fold), superoxide anion (O_2_^•−^, 1.5-fold), and hydrogen peroxide (H_2_O_2_, 2.33-fold) measured in the aortic rings increased. Blood serum antioxidant status (TAS, 1.6-fold), Cu (1.2-fold), Zn (1.1-fold), and the Cu/Zn ratio (1.4-fold) increased. An increase in Cu (1.12-fold) and the Cu/Zn ratio (1.09-fold) was also seen in the rats’ livers. Meanwhile, cyclooxygenase-1 (0.7-fold), cyclooxygenase-2 (0.4-fold) and glyceraldehyde 3-phosphate dehydrogenase (0.5-fold) decreased. Moreover, a negative correlation between Cu and Zn was found (r = −0.80) in rat serum. Supplementation with 200% Cu did not modify the isolated heart functioning. No significant difference was found in the body weight, fat/lean body ratio, and organ weight for either the heart or liver, spleen, kidney, and brain. Neither Fe nor Se, the Cu/Se ratio, the Se/Zn ratio (in serum and liver), heme oxygenase-1 (HO-1), endothelial nitric oxide synthase (eNOS), or intercellular adhesion molecule-1 (iCAM-1) (in serum) were modified. Supplementation with 200% of Cu potentiated pro-oxidant status and modified vascular contractility in middle-aged rats.

## 1. Introduction

In recent years, there has been a growing realization that the indispensable metallic micronutrients are of the utmost importance in various physiological processes. Copper is among the metallic elements found in this group. The significance of this phenomenon is readily apparent in both animal and human physiology. Copper, being an enzyme cofactor, plays a crucial role in the activation of various cuproenzymes (e.g., superoxide dismutase, ceruloplasmin, cytochrome-c oxidase). These cuproenzymes are responsible for facilitating important biological processes such as energy production, neuropeptide activation, iron metabolism, neurotransmitter synthesis, and connective tissue synthesis [1,2]. Moreover, copper indirectly plays a crucial role in angiogenesis, the maintenance of neurohormone balance, and the control of gene expression. It is also essential for proper brain development, pigmentation, and the functioning of the immune system [2]. The distinctive characteristics of copper, specifically its facile transition between the two oxidation states, copper(I) and copper(II), have resulted in its incorporation as a cofactor in numerous enzymatic processes involving oxidation and reduction reactions [3]. Copper, an indispensable trace element, can elicit oxidative stress by producing reactive oxygen species when present in an excessive amount [4]. Copper-facilitated lipid peroxidation has been observed in various in vivo and in vitro investigations [5,6]. An additional possible mechanism for the potentially harmful impacts of copper involves the formation of a copper-homocysteine complex, which has been hypothesized to elicit endothelial dysfunction and cause damage to blood vessels [7]. The mechanism of this damage is not fully understood, but it may be related to free radicals [8]. Based on the available data, the observed dose–response relationship between copper exposure and its potential toxic impact on vascular health suggests a linear trend. It is noteworthy that even at lower levels of average exposure in either water or food, which are prevalent in various regions worldwide, this toxic metal may exert adverse effects on the cardiovascular system [5]. Moreover, the direct or indirect involvement of copper imbalance in the etiology of various disorders has been well-documented and encompasses severe blood disorders, liver injury, cardiovascular diseases, and neurodegenerative alterations associated with dysfunctions in copper-dependent enzymes and proteins [9].

Oxidative stress and inflammation are recognized as crucial mechanisms behind endothelial dysfunction and arterial damage. These mechanisms establish a connection between risk factors such as hyperlipidemia, diabetes mellitus, and the development of vascular disease, arterial stiffness, and the aging processes [10]. The decline in physiological function associated with aging is a universal phenomenon that affects all organ systems. The vascular system exhibits a progressive pathological remodeling process characterized by stiffness, commonly linked to abnormalities in the extracellular matrix components of collagen and elastin. This phenomenon is somewhat contingent upon cellular senescence and growth halt [11]. 

Several limited-scale studies examining the effects of copper supplementation on cardiovascular disease risk factors in individuals without pre-existing health conditions have yielded minimal evidence suggesting any significant influence [12]. Given the limited number of investigations on the effects of elevated copper levels on the cardiovascular system and oxidative stress in middle-aged rats, coupled with the conflicting nature of certain findings, our objective was to thoroughly investigate this particular influence.

## 2. Materials and Methods

### 2.1. Drugs and Chemicals

Acetylcholine chloride (ACh), noradrenaline hydrochloride (NA), AH-6809 (No.: 33458-93-4), AL-8810 (No.: 246246-19-5), NS-398 (No.: 123653-11-2), and tranylcypromine-TCP (No.: 1986-47-6) were shipped from Sigma-Aldrich (St. Louise, MO, USA). Stock solutions (10 mM) of AH-6809, AL-8810, NS-398, TCP were dissolved in DMSO. Noradrenaline was dissolved in a NaCl + ascorbic acid (0.9% + 0.01% *w*/*v*) solution. The solvent concentration was of less than 0.01% (*v*/*v*). These solutions were stored at −20 °C, and appropriate dilutions were made in Krebs–Henseleit solution (KH in mM: NaCl 115; CaCl_2_ 2.5; KCl 4.6; KH_2_PO_4_ 1.2; MgSO_4_ 1.2; NaHCO_3_ 25; glucose 11.1) on the day of the experiment [13]. Copper carbonate of ≥99% purity was obtained from Poch (Gliwice, Poland).

### 2.2. Animals and Experimental Treatments

Three-week-old male Wistar rats (Cmdb: Wi CMDB from Charles River Laboratories, Sulzfeld, Germany, n/group = 12) were fed a standard rat diet (Diet A) for 6–7 months with 6.45 mg copper/kg of diet (100% of the recommended daily dietary quantity of copper) and 15.9 mg zinc/kg of diet, according to the recommendations of previous studies [13,14,15,16,17]. For the additional 8 weeks, rats from Group A were further supplemented with diet A. Meanwhile, rats from Group B received 12.9 mg copper/kg of diet (200% of the recommended daily dietary quantity of copper) and 15.9 mg zinc/kg of diet (Diet B) (see Table 1).

### 2.3. Vascular Reactivity Studies

Aortic rings were mounted in 5 mL chambers (Graz, Barcelona, Spain) under a pre-load tension of 1 g (FT20, TAM-A, Hugo Sachs Elektronik, March, Germany) as previously described [13]. High KCl (75 mM) and acetylcholine (10 µM) were used to check the functional integrity of aortic rings. Further, aortic rings were preincubated for 30 min with either the EP and DP receptor antagonist (AH-6809, 30 µM), the selective FP receptor antagonist (AL-8810, 10 µM), the selective cyclooxygenase-2 (COX-2) inhibitor (NS-398, 10 µM), the prostacyclin (PGI_2_) synthesis inhibitor (tranylcypromine, 10 µM) and contracted with noradrenaline (0.1 µM). The cumulative concentrations (CCs) of acetylcholine (0.1 nM–10 µM) were added into the incubation chambers to analyze vasodilatory response [13].

### 2.4. The Langendorff Heart

The Langendorff system with the ISOHEART software 73-0161 (Hugo Sachs Elektronik, March, Germany) was used for isolated heart studies as previously described [13].

### 2.5. Atomic Absorption Spectroscopy (AAS) for Cu, Zn, Fe, Se in Rat Serum and Liver

The concentration of elements was determined by AAS with flame atomization in acetylene–air flame at a wavelength of 248.3 nm (iron), 213.9 nm (zinc), and with electrothermal atomization at a wavelength of 324.8 nm (copper), and 196.0 nm (selenium), respectively, with a Zeeman background correction. Matrix modifiers were added for selenium analysis (palladium 1500 ppm, magnesium 900 ppm modifiers). The accuracy and precision of the methods were verified using certified reference materials (Seronorm Trace Elements Serum, Billingstad, Norway).

### 2.6. Total Antioxidant Status (TAS)

The Randox kit was used to measure the blood serum TAS by a spectrophotometric method.

### 2.7. ELISA Protocol

Commercial ELISA kits were used for COX-1, COX-2, heme oxygenase-1 (HO-1), endothelial nitric oxide synthase 3 (NOS3), glyceraldehyde 3-phosphate dehydrogenase (GAPDH), and intercellular adhesion molecule 1 (ICAM-1) determination in the blood serum (see Table S1). The absorbance in the ELISA test plate was measured by Thermo Scientific microplate readers (Varioskan LUX, Bremen, Germany) at a wavelength of λ = 450 nm.

### 2.8. Cu and Zn Determination in Rats’ Feed

Wet mineralization of samples was performed in concentrated HNO_3_ using the microwave technique in a closed system (Berghof, SpeedWave, Eningen, Germany). Zinc was determined by the AAS technique with atomization in an acetylene–air flame at a wavelength of 213.9 nm, and copper by the AAS method with electrothermal atomization in a graphite cuvette at a wavelength of 324.8 nm (Hitachi Z-2000, Tokyo, Japan).

### 2.9. Body Composition Analysis

Precise fat, fluid and lean tissue measurements on rats were performed with Bruker Minispec LF (Ettlingen, Germany) based on TD-NMR.

### 2.10. NO, O_2_^•−^ and H_2_O_2_ Detection

Diaminofluorescein (DAF), dihydroethidium (DHE) and 2′,7′-dichlorofluorescein diacetate (DCF) were used to evaluate the nitric oxide, superoxide anion and hydrogen peroxide levels in situ, as previously described [18]. Incubation was also conducted with the nuclear dye 4′,6-diamidino-2-phenylindole (DAPI, 10 µg/mL) as DAF + DAPI, DHE + DAPI and DCF + DAPI for 30 min in the oven at 37 °C. The fluorescence images were obtained with a LEICA (TCS ST2DM IRE2) laser scanning confocal microscope (568 nm and 410–475 nm for the DAPI dye).

### 2.11. Data Analysis and Statistics

Vascular contraction to noradrenaline (0.1 μM) was expressed in mg of tension; vascular relaxation to acetylcholine was expressed as a percentage of the contractile response to noradrenaline. The cumulative concentration–response curves (CCRCs) were analyzed by a log agonist vs. response (nonlinear regression model). The group comparison was performed by either a parametric (*t*-test) or non-parametric test (Mann–Whitney U-test or Kruskal–Wallis test). The number of animals varies due to technical reasons and material availability. CCRCs were analyzed by two-way ANOVA with Šídák’s multiple comparisons test. The results are expressed as the means ± SEM (for CCRCs) and means ± SD. The level of significance was set as * *p* ≤ 0.05.

## 3. Results

### 3.1. Copper and Zinc Concentration in Rats’ Feed (Atomic Absorption Spectroscopy—AAS)

In rats fed copper, the concentration was 7.83 ± 1.16 mg/kg of diet (in Group A) and increased to 17.86 ± 1.37 mg/kg of the diet (in Group B). Zinc was determined at similar levels of 25.59 ± 3.28 mg/kg (Group A) and 24.55 ± 3.58 mg/kg (Group B).

### 3.2. Animal Weight Gain and Body Composition (Time-Domain NMR)

The body weight gain, the weight of internal organs (heart, liver, spleen, kidney, brain), and the ratio of body fat to lean part (TD-NMR) did not change and were like those reported in previous studies [13,14] (see Table S2).

### 3.3. The Langendorff Heart

The values obtained from the isolated heart technique of the middle-aged rats (cardiac contractile strength and the heart rate) did not change between the groups (see Table S2). 

### 3.4. Vascular Contraction

**Figure 1:**
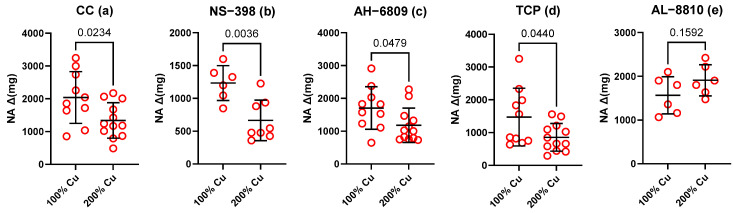
Vascular contraction to noradrenaline (NA, 0.1 µM) in the isolated thoracic arteries dissected from rats supplemented with 6.45 mg (100%—Group A) and 12.9 mg (200%—Group B) Cu/kg of diet for 8 weeks. Aortic rings were studied under the control conditions (**a**) or after preincubation with the selective cyclooxygenase-2 (COX-2) inhibitor (NS-398, 10 µM) (**b**), the EP and DP receptor antagonist (AH-6809, 30 µM) (**c**), the prostacyclin (PGI2) synthesis inhibitor (tranylcypromine, 10 µM) (**d**), the selective FP receptor antagonist (AL-8810, 10 µM) (**e**). Values are means ± SD, *p* ≤ 0.05 (*t*-test).

The vasoconstrictor response to high KCl (75 mM) did not differ between the groups (2.1 ± 0.05 g Group A vs. 2.2 ± 0.06 g Group B). However, noradrenaline (10 µM)-induced contraction decreased by 0.66-fold in the B Group (control conditions-CC) (see Figure 1a). Preincubation with NS-398 (0.5-fold, Figure 1b), AH-6809 (0.7-fold, Figure 1c), and TCP (0.6-fold, Figure 1d) decreased contraction similarly to CC, which points to the small effect of these factors on vascular contraction. In contrast, changes in vasoconstriction were not observed for AL-8810 (1.4-fold, Figure 1e), which points towards the FP receptors which modified this response (see Table 2).

**Figure 2:**
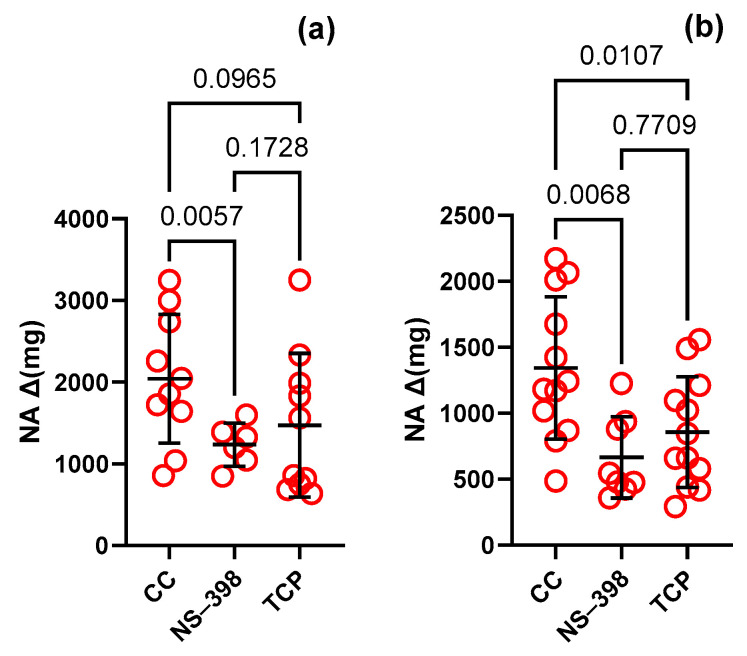
Vascular contraction to noradrenaline (NA, 0.1 µM) in the isolated thoracic arteries dissected from rats supplemented with 6.45 mg (100%—Group A) (**a**) and 12.9 mg (200%—Group B) (**b**) Cu/kg of diet for 8 weeks. Values are means ± SD, *p* ≤ 0.05 (two-way ANOVA with Tukey’s multiple comparisons test).

In the control group (Group A, Figure 2a), NS-398 and TCP decreased vascular contraction to NA compared to the control conditions by 0.6-fold and 0.7-fold, respectively. In the examined group (Group B, Figure 2b), contraction was decreased to a higher extent by 0.5-fold and 0.6-fold, respectively. Neither AH-6809 nor AL-8810 modified the contractile response to NA in Group A and Group B (see Table 2).

### 3.5. Vascular Relaxation

**Figure 3:**
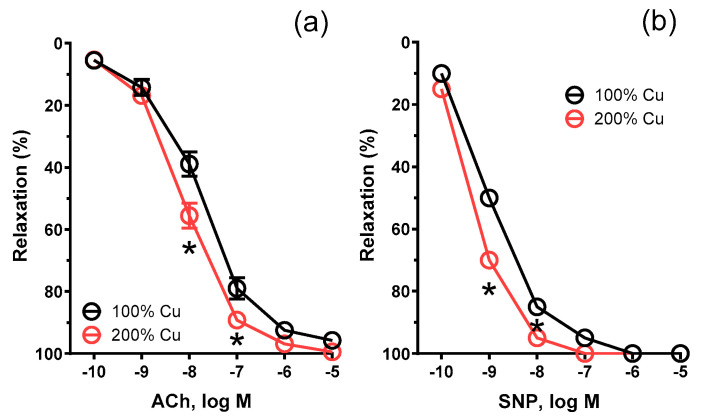
Vasodilation to cumulative concentrations of acetylcholine (ACh, 0.1 nM−10 µM) (**a**) and sodium nitroprusside (SNP, 0.1 nM−10 µM) (**b**) in the isolated thoracic arteries dissected from rats supplemented with 6.45 mg (100%—Group A) and 12.9 mg (200%—Group B) Cu/kg of diet for 8 weeks. Values are means ± SEM, n = 12, * vs. control diet (100% copper), *p* ≤ 0.05 (two-way ANOVA with Šídák’s multiple comparisons test).

The vasodilator response to acetylcholine (10–100 nM) and sodium nitroprusside was potentiated in Group B by 1.13-fold, *p* = 0.0154 (AUC), and 1.10-fold, *p* = 0.0348 (AUC), respectively (see Figure 3 and Table 3).

**Figure 4:**
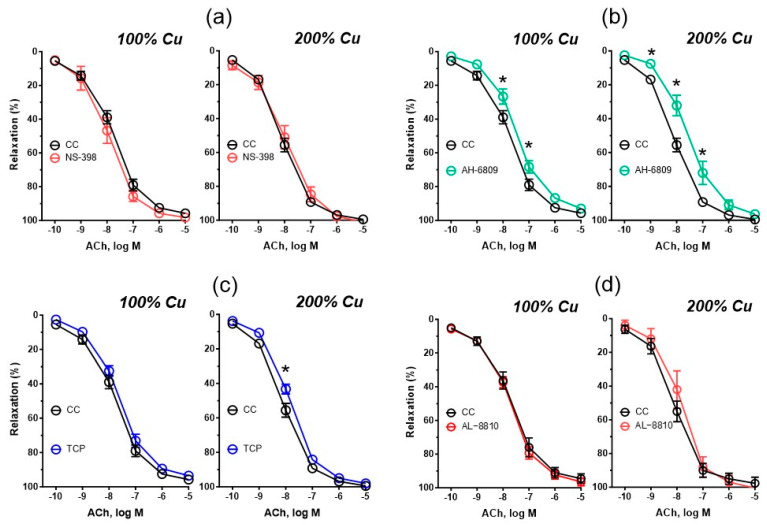
The cumulative concentration–response curves to acetylcholine (ACh, 0.1 nM–10 µM) in the isolated thoracic arteries dissected from rats supplemented with 6.45 mg (100%—Group A) and 12.9 mg (200%—Group B) Cu/kg of diet for 8 weeks. Aortic rings were preincubated (30 min) with the selective cyclooxygenase-2 (COX-2) inhibitor (NS-398, 10 µM) (**a**), the EP and DP receptor antagonist (AH-6809, 30 µM) (**b**), the prostacyclin (PGI2) synthesis inhibitor (tranylcypromine, 10 µM) (**c**), the selective FP receptor antagonist (AL-8810, 10 µM) (**d**). Values are means ± SEM, n varies, * vs. control conditions, *p* ≤ 0.05 (two-way ANOVA with Šídák’s multiple comparisons test). CC—control conditions.

Compared to the control conditions, preincubation with AH-6809 and TCP decreased vasodilation to acetylcholine in Group B by 0.8-fold, *p* = 0.001 and 0.9-fold, *p* = 0.0389, respectively. In the control group (Group A), a significant decrease was observed when aortic rings were pre-incubated with AH-6809 by 0.9-fold, *p* = 0.05 but not TCP. No significant difference was observed after preincubation with NS-398, AL-8810 or 1400W (see Figure 4 and Figure S2).

Compared to Group A, no significant difference in vasodilation was observed after preincubation with NS-398, AH-6809, and AL-8810; the same was not observed with TCP (see Figure S1). 

### 3.6. Total Antioxidant Status (TAS) in the Blood Serum

**Figure 5:**
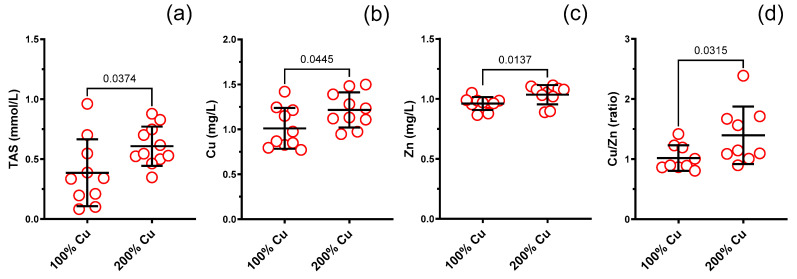
Total antioxidant status (TAS) (**a**), copper (Cu) (**b**), zinc (Zn) (**c**) and Cu/Zn molar ratio (**d**) measured in the blood serum of experimental rats supplemented with 6.45 mg (100%—Group A) and 12.9 mg (200%—Group B) copper/kg of diet for 8 weeks. Values are means ± SD, n varies, *p* ≤ 0.05 (*t*-test).

**Figure 6:**
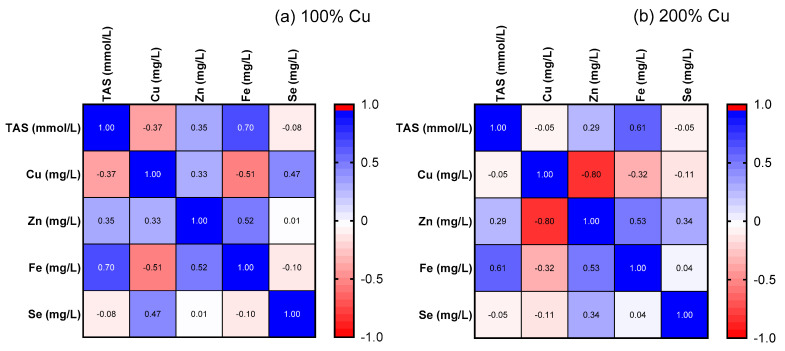
Correlation matrix. Pearson’s r correlation. Rats were supplemented with 6.45 mg (100%—Group A) (**a**) and 12.9 mg (200%—Group B) (**b**) Cu/kg of diet for 8 weeks. Positive correlation was found between TAS and Fe in both studied groups. Negative correlation was observed between Cu and Zn in the 200% copper group. Cu—copper, Zn—zinc.

TAS increased in Group B by 1.6-fold compared to the control group (Group A) (see Figure 5). A positive correlation was detected between TAS and Fe in both studied groups (r = +0.7, *p* = 0.024, Group A and +r = 0.61, *p* = 0.05, Group B) (see Figure 6).

### 3.7. Cu, Zn, Fe, Se in the Rat Serum (AAS)

Copper (1.2-fold), zinc (1.1-fold) and copper/zinc molar ratio (1.4-fold) increased together with copper supplementation (Group B) (see Figure 5). Neither iron (1.0-fold) nor selenium (1.0-fold) were changed (see Table S2).

Moreover, a negative correlation (r = −0.80, *p* = 0.006) was found between copper and zinc in Group B, which was not observed in Group A (r = 0.33, *p* = 0.3561) (see Figure 6).

### 3.8. Cu, Zn, Fe, Se in Rat Liver (AAS)

**Figure 7:**
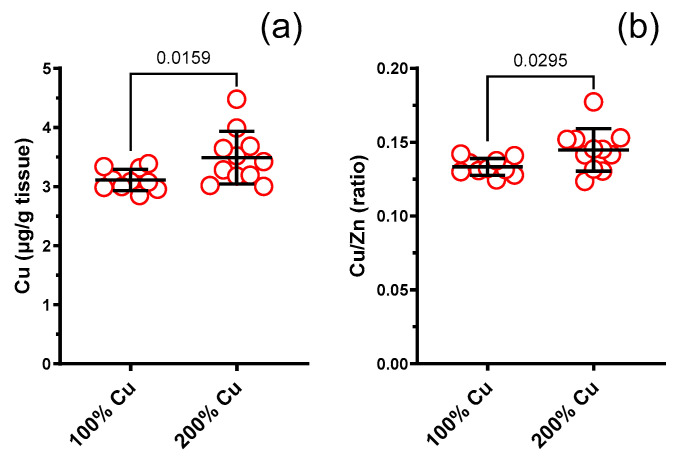
Copper (Cu) (**a**), and Cu/Zn molar ratio (**b**) measured in the liver of experimental rats supplemented with 6.45 mg (100%—Group A) and 12.9 mg (200%—Group B) Cu/kg of diet for 8 weeks. Values are means ± SD, n varies, *p* ≤ 0.05 (*t*-test).

Copper (1.12-fold) and the copper/zinc molar ratio (1.09-fold) increased together with copper supplementation (Group B) (see Figure 7). Neither zinc (1.05-fold) nor iron (1.05-fold) or selenium (1.0-fold) were changed (see Table S2).

### 3.9. ELISA Studies

**Figure 8:**
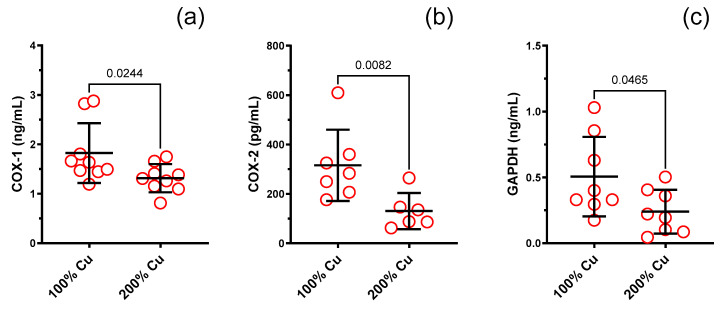
ELISA study. COX-1 (**a**), COX-2 (**b**) and GAPDH (**c**) measured in the blood serum of experimental rats supplemented with 6.45 mg (100%—Group A) and 12.9 mg (200%—Group B) Cu/kg of diet for 8 weeks. Values are means ± SD, n varies, *p* ≤ 0.05 (*t*-test).

COX-1 (0.7-fold), COX-2 (0.4-fold), and GAPDH (0.5-fold) decreased in Group B (see Figure 8 and Table 2). Neither HO-1 nor NOS3 or iCAM-1 were modified (see Table S2).

### 3.10. NO, O_2_^•−^ and H_2_O_2_ Detection

The fluorescence technique revealed increased tissue concentration of NO (2.08-fold), O_2_^•−^ (1.5-fold) and H_2_O_2_ (2.33-fold) with the increased Cu dose (see Table 2).

## 4. Discussion

The available literature lacks data on the effect of copper supplementation on aortic contractility and relaxation in the aging body; so, our research results fill this gap. It is worth emphasizing that the majority of feeding studies are conducted on young rats instead of aged animals, when pathological changes occur within the body. Aging processes modify the proper physiological vascular functioning, including increased vascular stiffness and decreased vascular response to noradrenaline and acetylcholine. In the presented study, increased copper intake resulted in a weaker vasoconstriction under the influence of noradrenaline. Similar to these results, copper at 10 and 16 µM (≈0.635 and 1.016 mg/L) has also been documented to inhibit phenylephrine-mediated contraction in a dose-dependent manner in isolated rings of rat thoracic aorta in vitro [19]. Another in vitro study revealed that copper pretreatment antagonized noradrenaline-induced vasoconstriction dependently and independently on the nitric oxide bioavailability, suggesting that the copper-induced effect was caused by a mechanism other than nitric oxide [20]. Moreover, these results have suggested that variations in the serum concentrations of copper might lead to changes in the alpha 1-adrenoreceptor-mediated vasoconstrictive response. It is worth mentioning that in our experimentation, supplementation increased the copper concentration in the blood serum (1.2-fold, from 1.011 mg/L to 1.216 mg/L), as well as in the liver (1.12-fold, from 3.110 µg/g to 3.492 µg/g). Afrin et al. reached different conclusions, as exposure to 6 µM copper(II) (≈0.381 mg/L) enhanced phenylephrine-induced contraction of rat aortic rings in organ baths. The authors concluded that both the increased intracellular calcium and reactive oxygen (ROS) and nitrogen species (RNS) may produce copper(II)-induced hypercontraction, contrary to the cyclooxygenase (COX)-mediated pathway, which has little effect on copper(II)-mediated aortic ring contraction [21]. Our results have shown that preincubation with the selective prostaglandin F (FP) receptor antagonist (AL-8810), made insignificant the vasoconstrictor response between the groups. This was contrary to COX-2 inhibitor (NS-398), the non-selective EP and DP1 receptor antagonist (AH-6809) and prostacyclin synthesis inhibitor (TCP), which did not change the observed differences in the noradrenaline-induced vasoconstriction of isolated aortic rings between the studied groups (100% vs. 200% Cu). This was reflected in the subsequent results on the increased participation of ROS in the aortic rings of supplemented rats, namely, nitric oxide (NO, 2.08-fold), superoxide anion (O_2_^•−^, 1.5-fold) and hydrogen peroxide (H_2_O_2_, 2.33-fold).

The discrepancies described between the in vitro studies are difficult to explain. However, the differences between in vitro and in vivo studies might be due to the fact that during prolonged supplementation, other mechanisms related to vascular remodeling and activation of different enzymes may occur, opposite to in vitro studies, which do not modify the vascular structure or proteins but modify the ROS and RNS formation. Another explanation of the described discrepancies is based on differences in copper administration, rat strain and animal age.

Aging processes induce the impairment of endothelium-dependent vasodilation [22]. Decreased endothelial nitric oxide synthase (eNOS) and endogenous nitric oxide bioavailability are key factors of endothelial dysfunction. Tian et al. observed an increased expression of inducible nitric oxide synthase (iNOS), which generates oxidative stress through the formation of the toxic oxidant molecule peroxynitrite (ONOO^−^), which leads to a further decrease in nitric oxide bioavailability in the vascular tissues of elderly rats (24–25 months old, weight 400–500 g, Sprague Dawley rats). Furthermore, this study reported that the application of a potent, selective iNOS inhibitor (1400W) enhanced the functionality of endothelium-dependent vasodilation in aged animals due to the attenuation of ONOO^−^ formation [22]. However, in our study, iNOS inhibition did not modify the vasodilator response to acetylcholine in both studied groups.

In our study (9–10-month-old middle-aged Wistar rats, weighing 471.1–588.1 g), the administration of an increased copper dose potentiated in a significant way the sensitivity of aortic rings to acetylcholine and sodium nitroprusside (SNP). Acetylcholine induces vasodilation of the smooth muscles in the vascular system by activating multiple pathways, including those dependent on and independent of either nitric oxide or prostanoids [23]. In contrast, SNP-induced vasodilation constitutes a NO-independent mechanism, since SNP is the exogenous donor of NO; so, the vasodilator response is not dependent on the impaired NO bioavailability. In line with the presented results, Lamb et al. showed that copper supplementation facilitates arterial relaxation by potentiating nitric oxide bioavailability in carotid aortic rings of New Zealand white rabbits fed with a cholesterol diet [24]. In another in vitro study, Wang et al. examined the direct impact of copper on the vasoreactivity of the rat mesenteric artery. The mesenteric rings exhibited vasodilation in a dose-dependent manner when exposed to copper chloride (the higher the copper concentration, the greater the relaxation). Nevertheless, this vasodilatory effect was significantly reduced when the mesenteric rings were pre-treated with L-NAME, an inhibitor of eNOS [20], which suggests that the nitric-oxide-dependent mechanism was involved in this process. 

In opposition to the findings of the aforementioned study, prolonged incubation of aortic rings with copper(II) sulfate at concentrations of copper(II) lower than 1 µM (≈0.0635 mg/L) led to a decreased vasodilatory reaction to acetylcholine [25]. In contrast to these results, it has been observed that the presence of ionically bonded copper did not impede the nitric-oxide-mediated responses. In another experiment, the implementation of a standard copper dose compared to a copper-deficient diet (no added copper into the mineral matrix) potentiated vasodilation to acetylcholine and increased noradrenaline-induced contraction, together with an increase in the blood plasma copper from 3.4 µM (≈0.22 mg/L) to 18.3 µM (≈1.16 mg/L), ceruloplasmin, lipid hydroperoxides (LOOH) and malondialdehyde (MDA). Meanwhile, there was a decrease in superoxide dismutase (SOD) and ferric reducing antioxidant power (FRAP) [14].

Another explanation of the observed potentiated changes in vasorelaxation to acetylcholine may be due to the fact that the aortic rings from our experimental group had a weaker response to noradrenaline both in the control group (control conditions) and after preincubation with NS-398, AH-6809 and TCP.

Interestingly, Majewski et al. described the advantageous administration of dietary resveratrol in conjunction with a standard copper dose, which manifested as enhanced vasodilation to acetylcholine and SNP, which, when together, constitute the mechanism independent on nitric oxide bio-availability, and decreased vasoconstriction to noradrenaline, along with reduced markers of lipid peroxidation in the blood plasma, reflected as decreased LOOH and MDA in supplemented rats (12 weeks of age Wistar rats) [15]. In the other study, the interaction between copper and homocysteine slowed down the endothelium-dependent arterial dilatation, which potentially facilitates the development of retinal vascular disease [26].

We have also investigated the COX pathway in which copper may exert an impact on the vasodilation of blood vessels in response to acetylcholine. During the process of preincubating aortic rings with various receptor inhibitors/antagonists (the selective COX-2 inhibitor NS-398, the non-selective EP1, EP2, EP3, and DP1 receptor antagonist AH-6809, and the selective FP receptor antagonist AL-8810), no statistically significant differences were observed in the vascular relaxation of precontracted aortic rings between the two analyzed copper groups. Interestingly, Nelson et al. in their study on the role of dietary copper found a possible involvement of copper deficiency in the reduction in prostacyclin secretion in the aortic rings [27]. In our study, arteries that were preincubated with the prostacyclin synthesis inhibitor tranylcypromine exhibited an increased vasodilator response to acetylcholine compared to the control group and a decreased vasodilator response compared to the control conditions, which indicates the involvement of the increased copper supplementation in the COX pathway and the increased participation of prostacyclin derived from COX-1, but not COX-2 in vasodilation, since the acetylcholine vasodilator response under the influence of the selective COX-2 antagonist (NS-398) was not modified. This points towards the prostacyclin pathway being involved in an increased vascular relaxation of 200% copper-supplemented rats. In previous studies, Majewski et al. demonstrated that prior exposure to inhibitors targeting both COX-2 and COX-1/2 reduced the vasodilatory response in copper nanoparticle-supplemented rats. Majewski et al. suggested that prostanoids derived from COX-2 play a significant role in the overall vasodilatory effect in copper-nanoparticle-fed rats [28], and this was due to the effect of nanoparticles rather than the copper ions themselves. Regrettably, the existing literature lacks additional references to the dietary ionic copper surplus in the COX pathway, leaving a notable dearth of research pertaining to the impact of elevated copper levels on vessels, as well as the underlying mechanisms behind this action.

Our findings have also revealed that an increased amount of dietary copper (200%) led to a reduction in the levels of COX-1, COX-2, and GAPDH within the blood serum of rats. No significant differences were observed regarding iCAM-1, HO-1, and eNOS when exposed to an excess of copper. Both COX-1 and COX-2, as pivotal enzymes in the cardiovascular system of arachidonic acid metabolism to prostaglandins, play a crucial role in a wide array of functions that can at times exhibit contrasting effects. The pro-thrombotic nature of the enzyme COX-2 in platelets is attributed to its ability to facilitate the synthesis of thromboxane. In contrast, it is noteworthy that the production of prostacyclin by COX-1 in endothelial cells exhibits antithrombotic properties. Additionally, within the kidney, COX plays a crucial role in regulating renal function and blood pressure [29]. A reduced concentration of COX-2 is the mechanism engaged in the control of enhanced oxidative stress when enzymes became depleted due to excessive exposure. Interestingly, Schuschke et al. reported an enhanced expression of COX-2 in response to insufficient dietary copper consumption [30], which may suggest that indeed copper excess may inhibit COX-2. On the other hand, a decrease in COX-1 isoform remains controversial, as it may have some adverse effects on the gastrointestinal tract and coagulation processes. 

Glyceraldehyde-3-phosphate dehydrogenase (GAPDH) is the enzyme essential for energy metabolism and cytoplasmic anaerobic glycolysis to produce ATP and pyruvate. GAPDH has many activities beyond energy metabolism. Increased GAPDH gene expression and enzymatic function are linked to cell proliferation and tumorigenesis, while increased oxidative stress impairs GAPDH catalytic activity and causes cellular aging and apoptosis [31]. This confirms the results of our studies, which indicate an increased presence of ROS and RNS in the aorta, and GAPDH in the blood. Khan et al. conducted a study to evaluate the baseline skin perfusion and brachial artery flow-mediated dilatation (FMD) while also exploring potential associations between vascular responses and blood GAPDH Ct levels. Higher Ct values were observed to correspond to reduced levels of GAPDH mRNA, indicating a positive correlation between elevated values of FMD, and diminished expression of GAPDH in the bloodstream [32].

Changes in the total antioxidant status suggest the presence of an increased oxidative stress and heightened vulnerability to oxidative harm [33]. In our study, the total antioxidant status of blood was increased, indicating a significant enhancement of the body’s ability to counteract oxidative damage. This outcome suggests that due to the presence of increased dietary copper, increased oxidative stress occurred. This was confirmed by the parallel increase in NO, O_2_^•−^ and H_2_O_2_, which was determined in the aortic rings. Certain investigators have suggested a noteworthy correlation between indicators of oxidative stress, such as elevated levels of NT (nitro-tyrosine) and modified TAS, and a progressive rise in dietary and serum copper concentrations [34]. The relationship between excess copper and oxidative stress remains inadequately explicated in the existing body of scientific literature.

In addition, we noticed significant differences between the two groups of supplemented rats with regard to an increased concentration of copper, zinc, and the copper/zinc ratio in the blood, with no alterations in the concentration of either selenium or iron. Neither the copper/selenium nor the selenium/zinc ratio were changed by copper supplementation, which, at least in part, might be explained by the fact that zinc increased only 1.1-fold in this study. Additionally, a negative correlation between the copper and zinc concentration was observed in the blood serum of 200%-copper-supplemented rats which was not seen in the control group (100% copper). Interestingly, this experimentation has revealed a significant positive correlation between the total antioxidant status (TAS) and the iron concentration in the blood serum of rats from both studied groups, even though the serum iron was not modified by the increased copper intake, contrary to the increased TAS.

The concentration of elements, especially the concentration of copper, does not always allow us to properly assess their possible deficiency in the body; so, it is better to determine their content in the liver, as this allows to take a closer look at the processes taking place in the body. However, only copper increased in the rat liver. Neither zinc nor iron or selenium changed. 

An elevated level of copper and decreased zinc represents a frequently encountered trace-metal imbalance [35]. Copper availability is subjected to numerous influencing factors, among which zinc supplementation emerges as a particularly noteworthy factor of clinical significance. The administration of zinc has been observed to stimulate the expression of the metal-binding protein known as metallothionein within the gastrointestinal tract, thereby leading a consequential reduction in the absorption of copper [36]. However, in this study, this was not the case since the rats were not supplemented with zinc. Although serum zinc increased in the entire group, the tendency within the group was a decrease in zinc with a concurrent copper increase. 

There were no observed statistically significant variations in body weight gain, fat, or lean body composition. Furthermore, no noticeable discrepancies were detected in organ weight for either the heart or liver, spleen, kidney, and/or brain. The findings of earlier research corroborate the information presented in this report [37]. In line with our findings, Filetti et al. demonstrated that animals in both the control group and those that received varying doses of copper showed no significant differences in weight at the beginning and at the end of the treatment [38]. This is contrary to Banach et al., who found that excess body weight relates to an increased concentration of copper [39]. Interestingly, changes were also observed in copper-deficient mice, which exhibited notable variations in organ weight in comparison to the control group. During copper deficiency, there was an observed increase in both liver and heart weight when compared to the control group. The mice that were deficient in copper exhibited a decrease in absolute brain weight, while kidney weight remained unchanged without any significant alterations [40]. The Langendorff technique (the isolated perfused heart assay) was also not modified. Interestingly, Filetti et al. suggested that copper administration over 4 weeks can impair cardiac contraction without altering vascular reactivity [38]. However, in this specific study, copper was injected via i.p. instead of given with food, and different concentrations were examined.

## 5. Conclusions

Our experimentation exposed that supplementation with 200% copper modified vascular contraction and relaxation, and this was due to increased ROS and prostaglandin formation, which modified the antioxidant status in middle-aged rats. It is imperative to conduct further investigations concerning the ramifications of elevated dietary copper on living organisms as a consequence of the escalating levels of environmental food and water contamination, as well as the presence of metabolic syndrome.

## Figures and Tables

**Table 1:** The experimental diet composition.

	(%)		g/kg of Diet	
Casein	20		200	
DL-methionine	0.3		3.0	
Cellulose	5.0		50	
Sucrose	10		100	
Rapeseed oil	2.0		20	
Lard	6.0		60	
Vitamin mixture	1.0		10	
Mineral mixture *	3.5		35 ^†^	
Choline chloride	0.2		2.0	
Corn starch	52 (to 100%)		520	
* Mineral mixture composition	% of mineral mixture		g/kg of diet	
Calcium carbonate (CaCO_3_)	35.7		12.495	
Single-basic potassium phosphate (K_2_HPO_4_)	19.6		6.86	
Potassium citrate (K_3_C_6_H_5_O_7_)	7.078		2.4773	
Sodium chloride (NaCl)	7.4		2.59	
Potassium sulfate (K_2_SO_4_)	4.66		1.631	
Magnesium oxide (MgO)	2.4		0.84	
Micro mix **	1.8		0.63 ^‡^	
Corn starch	21.362 (to 100%)		7.4767 (to 35.0 ^†^)	
** Micro mineral mixture composition	% of Diet A	% of Diet B	mg/kg of Diet A	mg/kg of Diet B
Iron citrate [16.7% Fe]	31.0	31.0	195.3	195.3
ZnCO_3_ [56% Zn]	4.5	4.5	28.35	28.35
MnCO_3_ [44.4% Mn]	23.4	23.4	147.42	147.42
CuCO_3_ [55.5% Cu]	1.85	3.7	11.655	23.31
KJ	0.04	0.04	0.252	0.252
Citric acid	39.21 (to 100%)	37.36 (to 100%)	247.023 (to 630 ^‡^)	235.368 (to 630 ^‡^)

Rats were fed a standard rat diet for 6–7 months (Diet A). For additional 8 weeks, rats were supplemented with diet A (Group A) and diet B (Group B). ^†^—refers to 35 g of mineral mixture, ^‡^—refers to 630 mg/kg of micro mineral mixture, * Mineral mixture composition, ** Micro mineral mixture composition.The rats were housed as previously described [13]. Food and tap water (from 20 years old plumbing with 0.35 mg copper/L) were given ad libitum. The blood was collected from the caudal vena cava of anesthetized animals. Intraperitoneal injections of ketamine (100 mg/kg BW) and xylazine (10 mg/kg BW) were used for anesthesia. During the experimentation period, two rats died from the control group (Group A), including one that died after anesthesia.

**Table 2:** Significant differences detected.

	100%—Group A *†			200%—Group B †					
	n	Mean	“Std. Deviation”	n	Mean	“Std. Deviation”	*t* Test	*p* Value	x-Fold
NA induced contraction Δ(mg)	9	2040	789.3	9	1342	539.6	Unpaired *t* test	0.0303	0.7
NS-398 Δ(mg)	6	1234	265.9	8	665.7	309.3	Unpaired *t* test	0.0036	0.5
AH-6809 Δ(mg)	10	1706	648.9	12	1181	521.3	Unpaired *t* test	0.0479	0.7
TCP Δ(mg)	10	1471	879.9	12	856.5	420.2	Unpaired *t* test	0.0440	0.6
AL-8810 Δ(mg)	6	1422	467.1	6	1938	453.1	Unpaired *t* test	0.1592	1.4
TAS (mmol/L) in rat serum	10	0.386	0.279	11	0.607	0.164	Unpaired *t* test	0.0374	1.6
Cu (mg/L) in rat serum	10	1.011	0.227	10	1.216	0.195	Unpaired *t* test	0.0445	1.2
Zn (mg/L) in rat serum	10	0.961	0.054	10	1.020	0.080	Mann–Whitney test	0.0137	1.1
Cu/Zn molar ratio in rat serum	9	1.018	0.211	9	1.396	0.479	Mann–Whitney test	0.0315	1.4
Cu (µg/g) in the liver	10	3.110	0.180	11	3.492	0.446	Mann–Whitney test	0.0159	1.12
Cu/Zn molar ratio in the liver	10	0.133	0.006	11	0.145	0.014	Mann–Whitney test	0.0295	1.09
COX-1 (ng/mL) in rat serum	9	1.823	0.607	9	1.315	0.285	Mann–Whitney test	0.0244	0.7
COX-2 (pg/mL) in rat serum	7	315.9	144.3	6	130.6	73.11	Mann–Whitney test	0.0082	0.4
GAPDH (ng/mL) in rat serum	8	0.506	0.302	8	0.240	0.166	Unpaired *t* test	0.0465	0.5
NO detection (DAF) in aortic rings	5	6212	1012	5	12,600	1209	Unpaired *t* test	0.0254	2.08
O_2_^•−^ detection (DHE) in aortic rings	6	4026	358.8	5	6064	1789	Unpaired *t* test	0.0238	1.5
H_2_O_2_ detection (DCF) in aortic rings	5	6023	897.0	5	14,104	1187	Unpaired *t* test	0.0187	2.33

n varies: one rat died after oral glucose tolerance test, and one rat died after ketamine injection from the control group *. Moreover, a few simple lab mistakes occurred (aorta rupture, blood coagulation or not enough blood) †.

**Table 3:** The influence of sodium nitroprusside (SNP) and the EP and DP receptor antagonist (AH-6809, 30 µM), the selective FP receptor antagonist (AL-8810, 10 µM), the selective cyclooxygenase-2 (COX-2) inhibitor (NS-398, 10 µM), and the prostacyclin (PGI2) synthesis inhibitor (Tranylcypromine, 10 µM) on the vasorelaxant effects to acetylcholine (ACh, 0.1 nM–10 µM) of thoracic arteries from Wistar rats supplemented with 6.45 mg (100%) and 12.9 mg (200%) Cu/kg of diet for 8 weeks.

	100% Cu—Group A				200% Cu—Group B			
	n	AUC	Emax (%)	pEC_50_	n	AUC	Emax (%)	pEC_50_
Control Conditions	8	275.2 ± 33.58	94.33 ± 1.722	7.740 ± 0.062	9	310.1 # ± 23.95	97.94 ± 1.213	8.074 # ± 0.043
NS-398	6	295.6 ± 29.72	97.09 ± 2.412	7.906 ± 0.087	7	306.8 ± 40.23	98.44 ± 2.213	7.917 ± 0.080
AH-6809	10	244.7 * ± 37.1	90.98 * ± 2.835	7.497 * ± 0.104	12	241.2 * ± 44.0	93.81 ± 2.311	7.597 * ± 0.079
Tranylcypromine	9	251.8 ± 46.85	91.79 ± 1.906	7.641 ± 0.069	10	287.4 *# ± 22.91	96.76 ± 1.021	7.859 ± 0.035
AL-8810	6	272.6 ± 20.18	94.91 ± 1.743	7.709 ± 0.063	6	247.4 # ± 56.66	89.81 ± 3.853	7.685 ± 0.140
1400W	6	267.7 ± 42.35	96.36 ± 1.693	7.772 ± 0.055	6	253.3 ± 40.27	95.83 ± 1.385	8.126 # ± 0.053
SNP	6	385 ± 83.93	98.88 ± 1.417	8.661 ± 0.234	6	422.5 # ± 67.84	99.92 ± 3.626	9.397 # ± 0.263

Values are based on the concentration–response curves. Data are expressed as means ± SD where n represents the number of animals, n varies, *p* ≤ 0.05 * vs. control conditions, # vs. the control Group A as determined by ANOVA followed by Tukey’s post hoc test.

## Data Availability

The original contributions presented in this study are included in the article/supplementary materials.

## Supplementary Materials

The following supporting information can be downloaded at: https://www.mdpi.com/article/10.3390/nu16081172/s1, Table S1: The ELISA kits used for the determination of antioxidant status in the present study; Table S2: Significant differences not detected; Figure S1: The cumulative concentration–response curves to acetylcholine (ACh, 0.1 nM–10 µM) in the isolated thoracic arteries dissected from rats fed with experimental diets. Aortic rings were incubated with NS-398, AH-6809, TCP and AL-8810. Values are means ± SEM, n = 12, * vs. control diet (100% copper), *p* ≤ 0.05 (two-way ANOVA with Šídák’s multiple comparisons test); Figure S2: The cumulative concentration–response curves to acetylcholine (ACh, 0.1 nM–10 µM) in the isolated thoracic arteries dissected from rats fed with experimental diets. Aortic rings were incubated with 1400W. Values are means ± SEM, n varies, * vs. control conditions, *p* ≤ 0.05 (two-way ANOVA with Šídák’s multiple comparisons test).

## References

[1] Prohaska J.R., Erdman J.W., Macdonald I.A., Zeisel S.H. (2012). Copper. Present Knowledge in Nutrition.

[2] Collins J.F., Ross A.C., Caballero B., Cousins R.J., Tucker K.L., Ziegler T.R. (2014). Copper. Modern Nutrition in Health and Disease.

[3] Fukai T., Ushio−Fukai M., Kaplan J.H. (2018). Copper transporters and copper chaperones: Roles in cardiovascular physiology and disease. Am. J. Physiol. Cell Physiol..

[4] Jomova K., Valko M. (2011). Advances in metal−induced oxidative stress and human disease. Toxicology.

[5] Chowdhury R., Ramond A., O’Keeffe L.M., Shahzad S., Kunutsor S.K., Muka T., Gregson J., Willeit P., Warnakula S., Khan H. (2018). Environmental toxic metal contaminants and risk of cardiovascular disease: Systematic review and meta−analysis. BMJ.

[6] Fry R.S., Ashwell M.S., Lloyd K.E., O’Nan A.T., Flowers W.L., Stewart K.R., Spears J.W. (2012). Amount and source of dietary copper affects small intestine morphology, duodenal lipid peroxidation, hepatic oxidative stress, and mRNA expression of hepatic copper regulatory proteins in weanling pigs. J. Anim. Sci..

[7] Kang Y.J. (2011). Copper and homocysteine in cardiovascular diseases. Pharmacol. Ther..

[8] Konukoğlu D., Serin O., Ercan M., Turhan M.S. (2003). Plasma homocysteine levels in obese and non−obese subjects with or without hypertension; its relationship with oxidative stress and copper. Clin. Biochem..

[9] Nunes K.Z., Fioresi M., Marques V.B., Vassallo D.V. (2018). Acute copper overload induces vascular dysfunction in aortic rings due to endothelial oxidative stress and increased nitric oxide production. J. Toxicol. Environ. Health A.

[10] Steven S., Frenis K., Oelze M., Kalinovic S., Kuntic M., Bayo Jimenez M.T., Vujacic−Mirski K., Helmstädter J., Kröller−Schön S., Münzel T. (2019). Vascular Inflammation and Oxidative Stress: Major Triggers for Cardiovascular Disease. Oxid. Med. Cell Longev..

[11] Guzik T.J., Touyz R.M. (2017). Oxidative Stress, Inflammation, and Vascular Aging in Hypertension. Hypertension.

[12] DiSilvestro R.A., Joseph E.L., Zhang W., Raimo A.E., Kim Y.M. (2012). A randomized trial of copper supplementation effects on blood copper enzyme activities and parameters related to cardiovascular health. Metabolism.

[13] Majewski M., Gromadziński L., Cholewińska E., Ognik K., Fotschki B., Juśkiewicz J. (2023). The Interaction of Dietary Pectin, Inulin, and Psyllium with Copper Nanoparticle Induced Changes to the Cardiovascular System. Nutrients.

[14] Majewski M., Ognik K., Juśkiewicz J. (2019). Copper nanoparticles modify the blood plasma antioxidant status and modulate the vascular mechanisms with nitric oxide and prostanoids involved in Wistar rats. Pharmacol. Rep..

[15] Majewski M., Ognik K., Juśkiewicz J. (2019). The interaction between resveratrol and two forms of copper as carbonate and nanoparticles on antioxidant mechanisms and vascular function in Wistar rats. Pharmacol. Rep..

[16] National Research Council (US) Subcommittee on Laboratory Animal Nutrition (1995). 2, Nutrient Requirements of the Laboratory Rat. Nutrient Requirements of Laboratory Animals.

[17] Majewski M., Ognik K., Juśkiewicz J. (2020). The antioxidant status, lipid profile, and modulation of vascular function by fish oil supplementation in nano-copper and copper carbonate fed Wistar rats. J. Funct. Foods.

[18] Majewski M., Klett-Mingo M., Verdasco-Martín C.M., Otero C., Ferrer M. (2022). Spirulina extract improves age-induced vascular dysfunction. Pharm. Biol..

[19] Yan M., Liu D.L., Chua Y.L., Chen C., Lim Y.L. (2001). Effects of micromolar concentrations of manganese, copper, and zinc on alpha1−adrenoceptor−mediating contraction in rat aorta. Biol. Trace Elem. Res..

[20] Wang Y.C., Hu C.W., Liu M.Y., Jiang H.C., Huo R., Dong D.L. (2013). Copper induces vasorelaxation and antagonizes noradrenaline−induced vasoconstriction in rat mesenteric artery. Cell Physiol. Biochem..

[21] Afrin F., Basir S.F., Khan L.A. (2022). Copper−promoted hypercontraction of rat aortic rings and its mitigation by natural molecules.

[22] Tian J., Yan Z., Wu Y., Zhang S.L., Wang K., Ma X.R., Guo L., Wang J., Zuo L., Liu J.Y. (2010). Inhibition of iNOS protects endothelial−dependent vasodilation in aged rats. Acta Pharmacol. Sin..

[23] Holowatz L.A., Thompson C.S., Minson C.T., Kenney W.L. (2005). Mechanisms of acetylcholine−mediated vasodilatation in young and aged human skin. J. Physiol.

[24] Lamb D.J., Tickner M.L., Hourani S.M., Ferns G.A. (2005). Dietary copper supplements modulate aortic superoxide dismutase, nitric oxide and atherosclerosis. Int. J. Exp. Pathol..

[25] Chiarugi A., Pitari G.M., Costa R., Ferrante M., Villari L., Amico−Roxas M., Godfraind T., Bianchi A., Salomone S. (2002). Effect of prolonged incubation with copper on endothelium−dependent relaxation in rat isolated aorta. Br. J. Pharmacol..

[26] Emsley A.M., Jeremy J.Y., Gomes G.N., Angelini G.D., Plane F. (1999). Investigation of the inhibitory effects of homocysteine and copper on nitric oxide−mediated relaxation of rat isolated aorta. Br. J. Pharmacol..

[27] Nelson S.K., Huang C.J., Mathias M.M., Allen K.G. (1992). Copper−marginal and copper−deficient diets decrease aortic prostacyclin production and copper−dependent superoxide dismutase activity, and increase aortic lipid peroxidation in rats. J. Nutr..

[28] Majewski M., Juśkiewicz J., Krajewska−Włodarczyk M., Gromadziński L., Socha K., Cholewińska E., Ognik K. (2021). The Role of 20−HETE, COX, Thromboxane Receptors, and Blood Plasma Antioxidant Status in Vascular Relaxation of Copper−Nanoparticle−Fed WKY Rats. Nutrients.

[29] Mitchell J.A., Kirkby N.S., Ahmetaj−Shala B., Armstrong P.C., Crescente M., Ferreira P., Lopes Pires M.E., Vaja R., Warner T.D. (2021). Cyclooxygenases and the cardiovascular system. Pharmacol. Ther..

[30] Schuschke D.A., Adeagbo A.S., Patibandla P.K., Egbuhuzo U., Fernandez−Botran R., Johnson W.T. (2009). Cyclooxygenase−2 is upregulated in copper−deficient rats. Inflammation.

[31] Nicholls C., Li H., Liu J.P. (2012). GAPDH: A common enzyme with uncommon functions. Clin. Exp. Pharmacol. Physiol..

[32] Khan F., Choong W.L., Du Q., Jovanovi’c A. (2013). Real−time RT−PCR Ct values for blood GAPDH correlate with measures of vascular endothelial function in humans. Clin. Transl. Sci..

[33] Young I.S. (2001). Measurement of total antioxidant capacity. J. Clin. Pathol..

[34] Bo S., Durazzo M., Gambino R., Berutti C., Milanesio N., Caropreso A., Gentile L., Cassader M., Cavallo−Perin P., Pagano G. (2008). Associations of dietary and serum copper with inflammation, oxidative stress, and metabolic variables in adults. J. Nutr..

[35] Osredkar J., Sustar N. (2011). Copper and zinc, biological role and significance of copper/zinc imbalance. J. Clinic Toxicol..

[36] McClain C.J., McClain M., Barve S., Boosalis M.G. (2002). Trace metals and the elderly. Clin. Geriatr. Med..

[37] Agunbiade J.A., Babatunde G.M. (1995). Copper and iron supplementation in the tropical environment effects on haematological measurements, organ weights, tissue copper and iron. Niger. J. Anim. Prod..

[38] Filetti F.M., Schereider I.R.G., Wiggers G.A., Miguel M., Vassallo D.V., Simões M.R. (2023). Cardiovascular Harmful Effects of Recommended Daily Doses (13 µg/kg/day), Tolerable Upper Intake Doses (0.14 mg/kg/day) and Twice the Tolerable Doses (0.28 mg/kg/day) of Copper. Cardiovasc. Toxicol..

[39] Banach W., Nitschke K., Krajewska N., Mongiałło W., Matuszak O., Muszyński J., Skrypnik D. (2020). The Association between Excess Body Mass and Disturbances in Somatic Mineral Levels. Int. J. Mol. Sci..

[40] Cunnane S.C., McAdoo K.R., Prohaska J.R. (1986). Lipid and Fatty Acid Composition of Organs from Copper−Deficient Mice. J. Nutr..

