# Supplementary material for: The Effect of an Elevated Dietary Copper Level on the Vascular Contractility and Oxidative Stress in Middle-Aged Rats
Source: Nutrients. 2024 Apr 15;16(8):1172. doi: 10.3390/nu16081172 (PMC11054332; doi:10.3390/nu16081172)
Supplement: Supplementary file 1 [file nutrients-16-01172-s001.zip › nutrients-2919286-supplementary.pdf]

Table S1. The ELISA kits used for the determination of antioxidant status in the presented study.

	Antigen	ELISA kit catalogue number	Manufacturer, country	Assay range (pg/ml)
1.	Rat PTGS1 ELISA Kit	orb566784	Biorbyt, UK	0.156–10 ng/mL<br>Intra–Assay: CV<8%<br>Inter–Assay: CV<10%
2.	Rat PTGS2 ELISA Kit	orb1199748	Biorbyt, UK	78.1–5000 pg/mL<br>Intra–Assay: CV<10%<br>Inter–Assay: CV<12%
3.	Rat HO–1 ELISA Kit	orb567452	Biorbyt, UK	0.156–10 ng/mL<br>Intra–Assay: CV<8%<br>Inter–Assay: CV<10%
4.	Rat NOS3 ELISA Kit	orb566738	Biorbyt, UK	15.625–1000 pg/mL<br>Intra–Assay: CV<8%<br>Inter–Assay: CV<10%
5.	Rat GAPDH ELISA Kit	orb567887	Biorbyt, UK	0.156–11 ng/mL<br>Intra–Assay: CV<8%<br>Inter–Assay: CV<10%
6.	Rat ICAM–1 ELISA Kit	RAB0221–1KT	Sigma–Aldrich, Germany	28.81–7000 pg/mL<br>inter–assay cv: <12%<br>intra–assay cv: <10%

Table S2. Significant differences not detected.

	100% – Group A			200% – Group B					
	n	Mean	"Std. Deviation"	n	Mean	"Std. Deviation"	t test	p value	x–fold (B/A)
Body weight begin (g)	12	492.4	32.84	12	497.6	33.75	Unpaired t test	0.7080	1.0
Body weight final (g)	11	530.3	39.80	12	536.5	34.54	Mann Whitney test	0.7859	1.0
Body weight gain (g)	11	37.16	27.51	12	38.89	42.29	Unpaired t test	0.9093	1.0
Fat (g)	11	92.52	23.31	11	84.19	22.83	Unpaired t test	0.4072	0.9
Fat (%)	11	17.25	3.224	11	15.85	4.231	Unpaired t test	0.3929	0.9
Lean (g)	11	323.1	16.21	11	329.5	25.44	Unpaired t test	0.4946	1.0
Lean (%)	11	61.06	2.273	11	61.96	3.158	Unpaired t test	0.4490	1.0
F/L ratio	11	0.285	0.063	11	0.258	0.076	Mann Whitney test	0.6063	0.9
Heart (g)	11	1.110	0.074	12	1.140	0.120	Unpaired t test	0.4810	1.0
Liver small + large part(g)	11	22.92	3.302	12	22.94	2.995	Unpaired t test	0.9886	1.0
Spleen (g)	11	0.882	0.183	12	0.900	0.209	Mann Whitney test	0.9879	1.0
Kidney (g)	11	2.476	0.262	12	2.576	0.531	Mann Whitney test	0.9279	1.0
Brain (g)	11	2.028	0.086	12	2.042	0.090	Unpaired t test	0.7151	1.0
Fe (mg/L)	10	2.317	0.449	11	2.349	0.391	Unpaired t test	0.8643	1.0
Se (ug/L)	10	361.2	42.17	11	362.2	66.01	Unpaired t test	0.9689	1.0
Cu/Se (ratio)	10	2.813	0.5421	12	3.702	1.620	Mann Whitney test	0.1335	1.3
Se/Zn (ratio)	10	0.377	0.048	11	0.356	0.067	Unpaired t test	0.4348	0.9
Zn (µg/g of liver tissue)	9	23.14	1.151	10	24.30	1.328	Unpaired t test	0.0589	1.1
Fe (µg/g of liver tissue)	9	107.1	13.71	10	114.3	25.01	Unpaired t test	0.4581	1.1
Se (µg/g of liver tissue)	9	0.7519	0.064	10	0.7694	0.082	Unpaired t test	0.6132	1.0
HO–1 (ng/mL)	10	1.606	0.539	11	1.979	0.962	Mann Whitney test	0.3867	1.2
NOS3 (pg/mL)	10	92.18	20.85	12	86.32	27.67	Unpaired t test	0.5749	0.9
iCAM (pg/mL)	10	70.99	16.80	10	97.60	23.50	Unpaired t test	0.9638	1.4

n varies: rat died and/or a simple lab mistake occurred

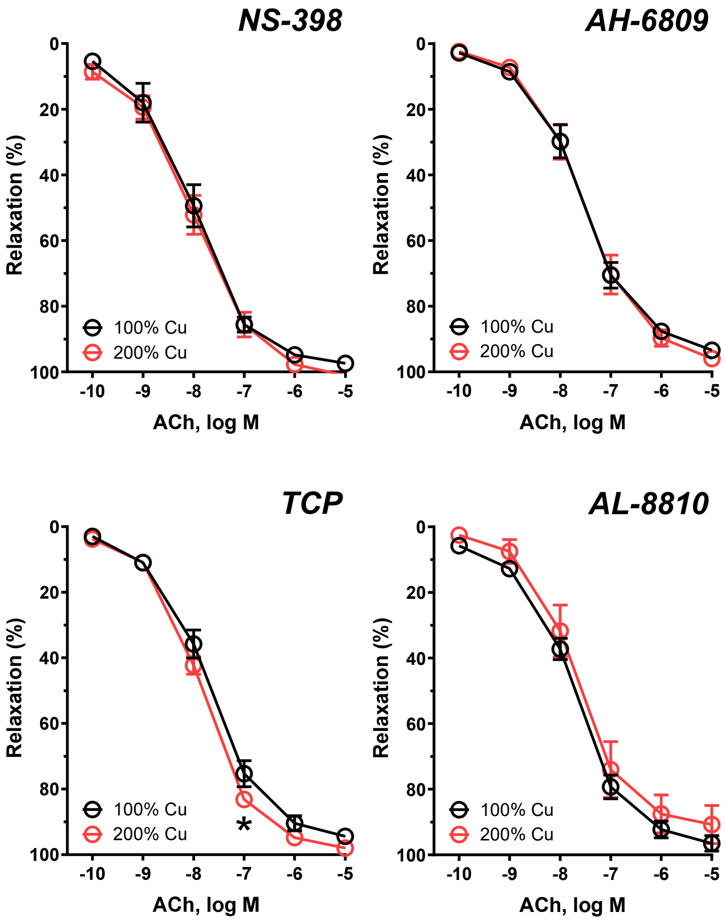

Figure S1. The cumulative concentration–response curves to acetylcholine (ACh, 0.1 nM–10 μM) in the isolated thoracic arteries dissected from rats fed with experimental diets. Aortic rings were incubated with NS–398, AH–6809, TCP and AL–8810. Values are means ± SEM, n = 12, * vs. control diet (100% copper), $p \leq 0.05$ (two–way ANOVA with Šídák's multiple comparisons test).

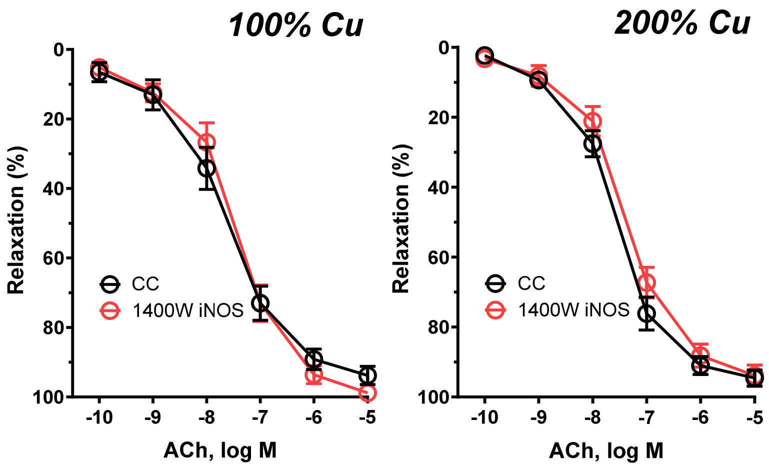

Figure S2. The cumulative concentration–response curves to acetylcholine (ACh, 0.1 nM–10 μM) in the isolated thoracic arteries dissected from rats fed with experimental diets. Aortic rings were incubated with 1400W. Values are means ± SEM, n = 12 (n varies), * vs. control conditions, $p \leq 0.05$ (two–way ANOVA with Šídák's multiple comparisons test).
